# Cancer-Associated Cachexia in the Era of Obesity

**DOI:** 10.3390/ijms262311626

**Published:** 2025-11-30

**Authors:** Joyce Cristina Ferreira de Resende, Márcia Fábia Andrade, Fabiana Amaral Ferreira, José Pinhata Otoch, Lívia Clemente Motta-Teixeira, Marilia Seelaender

**Affiliations:** 1 Cancer Metabolism Research Group, Laboratory of Experimental Surgery (LIM 26), Department of Surgery, Faculdade de Medicina da Universidade de São Paulo, HC-FMUSP, São Paulo 01246-903, Brazil; joyce.resende@usp.br (J.C.F.d.R.); marcia.andrade@usp.br (M.F.A.); fabiana.af@usp.br (F.A.F.); pinhata@usp.br (J.P.O.); livia.teixeira@fcmsantacasasp.edu.br (L.C.M.-T.); 2 Laboratory of Neuroplasticity and Behaviour, Department of Physiological Sciences, Santa Casa de São Paulo School of Medical Sciences, São Paulo 01224-001, Brazil

**Keywords:** cancer, cachexia, obesity, inflammation, adipose tissue

## Abstract

Obesity and cancer cachexia represent opposite ends of the body mass index (BMI) spectrum. However, despite this apparent dichotomy, both conditions share critical metabolic alterations, primarily driven by inflammation, metabolic alterations and changes in adipose tissue biology. Obesity is characterised by chronic low-grade inflammation and increased fat storage, while cancer cachexia involves severe weight loss, muscle atrophy, and lipolysis, with inflammation playing a central role in both conditions. Inflammatory cytokines such as TNF-α and IL-6 are increased in both scenarios, contributing to metabolic dysregulation and systemic energy imbalance. This review explores the shared inflammatory and metabolic pathways underlying obesity and cancer cachexia, with particular regard to the role of white adipose tissue (WAT). Moreover, we intend to highlight the importance of understanding the common mechanisms for the development of more effective therapeutic strategies for managing these complex metabolic disorders.

## 1. Introduction

Chronic non-communicable diseases (NCDs) represent the leading cause of mortality worldwide and constitute one of the greatest challenges in contemporary public health. Collectively, diseases such as cardiovascular disease (CVD) and cancer are responsible for approximately 70 percent of all global deaths [1]. Obesity is another condition adding as a risk factor for NCDs, markedly contributing to decreased life expectancy (up to 5 to 20 years overall decrease), depending on the severity of the condition and the presence of comorbidities [2,3,4]. Obesity is characterised, among other factors, by the excessive accumulation of body fat. It is associated with an increased frequency of several types of cancer, including those of the breast, colon, rectum, pancreas, and ovary [5,6,7,8,9,10,11]. In addition, obesity also augments the probability of tumour recurrence and mortality among cancer survivors [4]. In contrast, one of the most conspicuous and frequent hallmarks of cancer progression is involuntary weight loss, which is associated with the presence of the paraneoplastic syndrome of cachexia [12]. Cachexia, a multifactorial disorder, involves loss of skeletal muscle mass (muscle atrophy) and, frequently, pronounced adipose tissue loss, inflammation affecting multiple organs and tissues, as well as robust alterations of the major metabolic pathways [13]. Nutritional support, whether in conventional, enteral or parenteral forms, is unable to reverse cachexia [14]. The complex nature of this cancer comorbidity makes effective treatment a challenging issue [15,16,17]. The plethora of cancer-related cachexia (CC) symptoms include asthenia, loss of appetite, nausea, energy imbalance, wasting, and neuroendocrine disruption [18]. Around 50 to 80 percent of patients with advanced cancer develop the syndrome, with a higher incidence in patients with pancreatic, gastroesophageal, colorectal, and head and neck tumours [15,19,20]. According to the recent review by Morena da Silva et al. (2023), approximately 20 to 40% of cancer deaths are directly related to cachexia [21].

Interestingly, despite representing opposite extremes of the body mass index (BMI) spectrum, cancer cachexia and obesity share underlying inflammatory mechanisms that lead to major metabolic disruption in both scenarios. This review explores these mechanisms, focusing on two key aspects common to both conditions: inflammation and alterations in adipose tissue biology.

## 2. Inflammation: The Central Orchestrator

Inflammation is an essential physiological response to restore homeostasis in the face of harmful stimuli; however, when the inflammatory response becomes persistent or excessive, it may become chronic and negatively impact the organism. Disruption of energy metabolism, for instance, is one of the key factors associated with chronic inflammation in both cancer-related cachexia and in obesity [22].

One of the causes of cachexia is the impairment of normal tissue function caused by pro-inflammatory cytokines released at greatly augmented, non-physiological concentrations by tumours and subsequently, different cells and organs of the host. Tumour growth triggers, per se, an inflammatory response, which induces the recruitment and activation of immune cells such as macrophages, T lymphocytes and neutrophils within the tumour microenvironment, and the inflammatory status is communicated to peripheral tissues, which also develop a local inflammatory response, contributing to the systemic production of pro-inflammatory cytokines [23]. Among the factors secreted both by the tumour and the peripheral organs/tissues, those reported to be consistently and frequently changed in cachexia, are tumour necrosis factor-alpha (TNF-α), interleukin-1 (IL-1), interleukin-6 (IL-6), and growth differentiation factor 15 (GDF15), all of which promote muscle degradation and insulin resistance (IR) [13,24,25].

Macrophages and T lymphocytes are activated in response to the presence of tumours and, by releasing cytokines such as TNF-α, IL-6 and interferon-γ (IFN-γ), promote an inflammatory response that not only intensifies local inflammation at different sites, but is also involved in protein catabolism, leading to the loss of both fat and muscle tissue [26,27]. The combination of these factors creates a chronic proinflammatory environment that drives the process of tissue degradation and accelerates the loss of lean mass [28,29].

The detection of pathogen-associated molecular patterns (PAMPs) and cell damage-associated molecular patterns (DAMPs) by pattern recognition receptors (PRRs), notably Toll-like receptors (TLRs) on immune cells such macrophages and T lymphocytes, also triggers additional inflammatory responses [16,23]. This receptor-mediated inflammation exacerbates cachexia and its associated metabolic disorders [30]. Consequently, the up regulation of these inflammatory factors in both tumour microenvironment and peripheral tissues, leads to systemic inflammation, thereby compromising metabolic homeostasis. Several studies have demonstrated these effects in the liver, heart, brain, adipose depots, and muscle tissue [31,32]. These alterations not only contribute to weight and muscle mass loss but may also increase basal metabolic rate in some patients, aggravating the catabolic state [33], which is characteristic of the paraneoplastic syndrome. It is noteworthy that despite 40% to 60% of cancer patients being overweight, cachexia is a frequent comorbidity, and thus, it is considered independent of previous body mass index (BMI) [34,35].

In cachexia, mitochondrial dysfunction plays a central role by impairing cellular bioenergetics and increasing the production of reactive oxygen species, thereby contributing to a sustained inflammatory environment. This mitochondrial stress promotes the activation of both systemic and local inflammatory pathways, facilitating immune cell infiltration into tissues and exacerbating the loss of muscle and adipose tissue that characterises the syndrome [15,36,37].

Obesity is characterised by excessive fat accumulation and, similarly to cachexia, chronic low-grade systemic inflammation, which predisposes individuals to various chronic conditions, including the metabolic syndrome [38]. The chronic inflammatory state leads to changes in the adipose tissue, liver, pancreas, hypothalamus, and in the skeletal muscle [39,40,41,42]. The main inflammatory players in obesity are pro-inflammatory cytokines such as TNF-α, IL-6, and C-Reactive Protein (CRP), as well as adipokines such as leptin, adiponectin, resistin, among others secreted by adipose tissue [43,44]. These are the adipokines, factors secreted by the adipose tissue, and are also robustly altered in cachexia [45,46].

In addition to these well-established mechanisms, recent evidence highlights the critical roles of endoplasmic reticulum (ER) stress and mitochondrial dysfunction in the perpetuation of obesity-related inflammation. Metabolic overload in obesity leads to the accumulation of misfolded proteins within the ER, triggering the unfolded protein response (UPR). Chronic UPR activation promotes inflammatory signalling via NF-κB and the NLRP3 inflammasome, thereby exacerbating cytokine production and systemic inflammation [47,48].

Concurrently, mitochondrial stress resulting from excessive nutrient flux and oxidative damage contributes to the release of mitochondrial DNA (mtDNA) into the cytosol. mtDNAs act as damage-associated molecular patterns (DAMPs), which are recognised by cytosolic receptors such as cyclic GMP–AMP synthase (cGAS), leading to activation of the cGAS–STING pathway and induction of type I interferon responses [49]. Moreover, mtDNA can also directly activate the NLRP3 inflammasome, further sustaining the inflammatory milieu characteristic of obesity [50].

These cellular stress pathways—namely endoplasmic reticulum stress and mitochondrial dysfunction—not only amplify systemic inflammation but also create a pro-inflammatory tissue microenvironment that favours the recruitment and activation of immune cells. In particular, the dysregulated secretion of adipokines and cytokines from stressed adipocytes promotes chemotactic signals that drive the infiltration of immune cells into adipose tissue. This immune cell recruitment, especially of macrophages, plays a central role in sustaining obesity-associated inflammation and its metabolic consequences [51,52].

The infiltration of immune cells into the adipose tissue, especially macrophages, contributes to obesity-associated systemic inflammation. Adipose tissue-infiltrating macrophages make up 5 percent of the cells in the adipose tissue in normal-weight individuals. However, this figure increases dramatically to 50 percent in obese individuals, suggesting an important role for macrophage infiltration in obesity and its associated complications [53,54]. In individuals with normal weight, adipose tissue macrophages (ATMs) predominantly exhibit an anti-inflammatory M2 phenotype, characterised by the secretion of IL-10, IL-4, and TGF-β However, in individuals with obesity, an imbalance between M1 and M2 macrophages occurs, with a shift towards a pro-inflammatory (M1) state. This leads to the release of cytokines such as TNF-α, IL-6, and IL-1β, which drive inflammation, IR, and lipid metabolism dysregulation [55,56]. Additionally, T lymphocytes, particularly CD8+ and CD4+ Th1 cells, infiltrate visceral adipose tissue (VAT), further exacerbating inflammation and contributing to impaired glucose homeostasis. Other immune cells, including B lymphocytes, neutrophils, and dendritic cells, amplify the inflammatory response. This immune cell infiltration sustains a chronic inflammatory state, disrupting metabolism by increasing lipolysis and the release of fatty acids, thereby exacerbating IR [53,56,57]. Immune cell infiltration in the pancreas also disrupts pancreatic β cell function, impairing insulin production and contributing to type 2 diabetes [31,58,59]. Additionally, inflammatory cytokines affect endothelial cells, leading to endothelial dysfunction, which increases the risk of CVD such as hypertension and atherosclerosis [60,61,62]. It is plausible to postulate that obese individuals, already experiencing chronic low-grade inflammation, may be at an even higher risk of metabolic disturbances driven by cancer-associated systemic responses [63]. Similarly, during cachexia, infiltrating immune cells in various organs are a common finding, as we shall discuss ahead.

## 3. Adipose Tissue: A Regulator of Inflammation and Metabolism

Adipose tissue biology, can vary significantly among individuals due to several factors, including genetics, metabolism, hormonal regulation, diet, physical activity, and environmental/behavioural influences. Such variations are found in regard to fat pad anatomical distribution, adipocyte and vascular stromal fraction size, number and volume. metabolic activity, extracellular matrix and overall function [64,65,66]. Adipose tissue (AT) is composed of different types of cells that interact dynamically, leading to the secretion of various cytokines, chemokines, and hormones, thereby contributing to its endocrine function [67]. Approximately one-third of the cells present in white adipose tissue (WAT) are adipocytes, while the remainder consists of fibroblasts, endothelial cells, stromal cells, immune cells, and pre-adipocytes [57].

Adipocytes are highly specialised cells for the storage of triglycerides, a feature resulting from their ability to efficiently perform the esterification of free fatty acids into triglycerides which are then stored within lipid droplets. Although these characteristics are well established for one (white) of the many types of adipose tissue, they may vary greatly when different types are regarded. Adipose tissue may be very roughly divided into two main categories: white adipose tissue (WAT) and brown adipose tissue (BAT). WAT is specialised in energy storage, whereas BAT is thermogenic and mitochondria-rich, essential for energy dissipation and heat production [68,69]. Additionally, other types of adipocytes have been identified, including beige or brite adipocytes, which can be induced in WAT depots and possess thermogenic capacity; pink adipocytes, are involved in milk production; and perivascular adipocytes, which regulate vascular function [70,71,72,73]. Given its role in energy metabolism and inflammation, WAT will be our focus throughout this review.

The WAT plays a pivotal role in the storage of excess energy following meals and in the mobilisation of lipids during fasting, thereby meeting energy requirements of other tissues. Its signalling products are also essential for inter-tissue communication with organs including the liver, muscle, and the central nervous system, facilitating the integration of metabolic signals in response to the body’s energy demands, body composition, and appetite regulation, which are also under the influence of environmental factors [74,75]. WAT is capable of producing and releasing a variety of cytokines, such as TNF-α, IL-6, IL-1β, as well as of adipokines, such as leptin, adiponectin and visfatin, all of which are involved in inflammatory modulation [76,77], playing essential roles in the regulation of appetite, inflammatory and immune function, glucose and lipid metabolism, cardiovascular homeostasis, and reproduction, among other processes [78,79].

Beyond its endocrine and immunomodulatory roles, WAT is functionally and anatomically diverse, with distinct depots exhibiting specific metabolic and inflammatory properties. The distribution of adipose tissue, particularly the distinction between visceral (vWAT) and subcutaneous (sWAT) depots, plays a crucial role in energy homeostasis and disease states. Subcutaneous adipose tissue accounts for the majority of body fat, with significant depots in the abdominal, subscapular, gluteal, and femoral regions. Visceral adipose tissue includes intraperitoneal, retroperitoneal, mediastinal, gonadal, and pericardial depots [75,80]. This distinction is particularly relevant in the context of metabolic and inflammatory conditions. Patients with cancer cachexia present an increase in lipolysis in association with systemic inflammation [81]. Conversely, people with obesity frequently show low-grade chronic inflammation and ectopic accumulation which is characterised by fat deposition in tissues other than the adipose tissue [82].

## 4. Obesity and Inflammation

Obesity leads to the expansion of WAT through both hyperplasia (increased number of adipocytes, by recruitment of pre-adipocyte population) and hypertrophy (increased adipocyte size) to accommodate the increased demand for lipid storage and respond to hyperinsulinemia, which promotes lipogenesis [83,84]. The expansion of each adipose depot is highly specific and varies within the Mammalia class [85]. This process of adipose expansion, in turn, can lead to the accumulation of extracellular matrix proteins in the white adipose tissue, resulting in fibrosis, which is associated with a pro-inflammatory phenotype in human obesity [86,87].

Some studies suggest that the distribution and proportion of subcutaneous (sWAT) and visceral (vWAT) adipose tissue are critical factors in the development of IR [88,89,90,91]. Specifically, a higher proportion of sWAT relative to vWAT is associated with a lower likelihood of developing IR, whereas increased vWAT is linked to greater inflammatory responses and immune cell infiltration, particularly of M1 macrophages, which, as mentioned before, are associated with the production of pro-inflammatory cytokines [92,93,94,95]. On the other hand, sWAT does not contribute to the same extent to inflammation or lipotoxicity, suggesting that it may play a less detrimental role in metabolic dysfunction compared to vWAT [96]. IR is closely related to the balance of adipokines, particularly to the adiponectin/leptin ratio, which serves as a crucial marker of adipose tissue dysfunction and is inversely correlated with BMI [97,98,99,100].

The altered dynamics of leptin and insulin in obesity underscore the distinct roles of vWAT and sWAT in metabolic health [101]. Despite its usual smaller volume, vWAT shows a higher density of immune cells, resulting in increased production of pro-inflammatory cytokines such as IL-6 and TNF-α, which contribute to IR [102]. Leptin, a cytokine with inflammatory properties that exacerbates inflammation and metabolic dysfunction, is secreted by vWAT in higher proportions when compared to sWAT [103,104]. Leptin acts as a potent pro-inflammatory mediator, promoting the activation of immune cells and the production of additional inflammatory cytokines, thereby amplifying the inflammatory state within visceral fat depots. In contrast, sWAT is regarded as a more neutral fat depot, characterised by a lower presence of pro-inflammatory immune cells and greater secretion of adiponectin, a key adipokine with protective effects against IR and inflammation [105]. Research shows that sWAT is the primary source of circulating adiponectin, while adiponectin secretion from vWAT tends to decrease with increasing BMI and central obesity [101]. This imbalance marked by high leptin and reduced adiponectin levels illustrates the divergent roles of vWAT and sWAT in metabolic health and is associated with higher cardiometabolic risk, particularly in individuals with central fat accumulation. Thus, the specific anatomical distribution of adipose tissue in the human being plays a critical role in determining metabolic outcomes [106,107].

The increased leptin secretion of leptin by vWAT is also linked to the hormone’s role in the appetite regulation. Leptin is a key signal to the brain that transmits information regarding the body’s energy reserves. It crosses the blood–brain barrier and acts in the arcuate nucleus of the hypothalamus, where it modulates energy balance and appetite [108,109,110]. In this hypothalamic region, leptin influences two major neuronal populations: one that secretes neuropeptide Y (NPY) and agouti-related protein (AgRP), which stimulate appetite (orexigenic stimulus), and another that releases proopiomelanocortin (POMC), which suppresses appetite and decreases metabolic rate (anorexigenic stimulus). Leptin plays a well-established role in body weight regulation by inhibiting orexigenic signals and stimulating anorexigenic pathways. However, in obesity, augmented circulating leptin levels frequently fail to exert the typical anorexigenic effects, a phenomenon known as leptin resistance. This resistance is particularly pronounced in individuals with chronic inflammation and metabolic dysfunction, as observed in type 2 diabetes and other obesity-related conditions [111,112]. As a result, appetite dysregulation, hyperphagia, and weight gain occur [113]. The diminishing effectiveness of leptin in suppressing appetite underscores the complex interplay between neuronal pathways and highlights the intricate relationship between adipose tissue and the progression of obesity [114,115,116].

The notion that the cellular composition of adipose tissue changes in obesity, completely modifying tissue metabolism and function is now well established. Therefore, it is not merely the contribution of individual adipocytes that matter but rather the interactions between various cell types and local paracrine signalling that drive significant increases in cytokine secretion from adipose tissue in obesity. The literature on the subject is vast and it is not the role of this review to comprehensively address the WAT in obesity.

## 5. Cachexia and WAT Inflammation

In cancer cachexia, white adipose tissue (WAT) undergoes profound phenotypic and metabolic changes, deviating from normal healthy patterns These include marked alterations in the efficient storage of fat, in the balance between lipogenesis and lipolysis, and in the response to insulin and hormonal signals regulating energy metabolism [117,118]. In cachexia, these processes are disrupted, leading to adipocyte atrophy [16,32,119]. The impairment of WAT biology is driven by multiple mechanisms, including increased lipolysis, enhanced lipid utilisation, and decreased adipogenesis and lipogenesis, all of which play a key role in the progression of the paraneoplastic syndrome [120,121,122]. In addition, several proinflammatory factors, such as TNFα, IL-1β, and IL-6, are increased in WAT during cachexia, as a result of both adipocyte secretion and immune cell infiltration. Previous studies by our group have shown that the release of these factors into the circulation by the tumour and other compartments leads to immune cell recruitment to WAT and local inflammation [123]. The tissue, in turn, increases the synthesis and release of inflammatory factors, with paracrine and endocrine effects. Among the various inflammatory cytokines secreted by WAT, TNF-α stands out as a regulator of IR and induces apoptosis in preadipocytes and adipocytes [124]. Along with other cytokines and hormones, TNF-α contributes to the metabolic alterations observed in cachectic patients [125,126].

As commented on earlier, tumours actively secrete a variety of cytokines and pro-inflammatory mediators, including IL-6, TNF-α, and IL-1β, which can reach the adipose tissue and trigger the recruitment of immune cells, such as macrophages and T cells, leading to a chronic inflammatory state [127,128]. This causes the secretion of additional inflammatory factors by the AT itself, including leptin, resistin, and monocyte chemoattractant protein-1 (MCP-1), which further exacerbate systemic inflammation and metabolic dysfunction associated with cachexia [129]. The inflammatory response also induces extracellular matrix remodelling, with the accumulation of elastic fibres, type I, II, and VI collagen, and fibronectin, leading to fibrosis and impaired adipose tissue function [130,131].

Therefore, the crosstalk between the tumour and the AT establishes a vicious cycle in which tumour-derived cytokines drive AT inflammation and dysfunction, which in turn amplifies the systemic inflammatory state characteristic of cachexia, contributing significantly to metabolic derangements and general tissue wasting [127,128].

Recent findings indicate that adipose tissue loss occurs earlier than other traditional hallmarks of cachexia, with fat depletion happening more rapidly and earlier than the loss of lean mass. In this context, the location of WAT, as in obesity, is important in the scenario [132]. Depletion of sWAT is associated with worsened prognosis in cancer cachexia, with increased energy expenditure, reduced quality of life, and reduced survival time. In contrast, vWAT depletion has no negative impact on prognosis or survival [133]. In a previous study by our group, we demonstrated that cachectic patients present a decreased leptin/adiponectin ratio compared to patients with stable weight. The mRNA expression of adiponectin increased in sWAT of cachectic patients, while vWAT expression remained unchanged, suggesting that subcutaneous adipose tissue is the primary contributor to plasma adiponectin alterations [123].

Radiodensity refers to the ability of a tissue to attenuate X-rays, which is commonly measured using computed tomography (CT) scans. Higher radiodensity values in subcutaneous adipose tissue indicate a higher fat content and denser tissue structure, which may reflect changes associated with tumour-induced metabolic alterations [134,135].

Recently, Sun and collaborators [136] demonstrated that higher subcutaneous adipose tissue radiodensity is linked to increased tumour metabolic activity and poorer survival outcomes in patients with non-small-cell lung cancer, highlighting its potential role in the diagnosis and assessment of cachexia progression. Cancer-associated cachexia also leads to altered secretion of adipokines. Although the influence of the dysregulation of these molecules on cachexia is not yet fully understood, it is common for cachectic patients to have altered levels of adipokines in the blood, such as of leptin, adiponectin and resistin [132]. Studies have shown that leptin levels are significantly reduced in cachectic cancer patients compared to healthy, non-cachectic individuals [137,138,139]. In cancer patients, leptin correlates with adiposity indices and, unlike in healthy individuals, is positively associated with appetite; however, low leptin levels in cachectic patients are likely a consequence of reduced fat mass, partially caused by anorexia [140].

Anorexia, characterised by loss of appetite, is a common symptom of cancer—cachexia is associated with alterations in neurotransmitters and neuropeptides in the central nervous system (CNS), particularly in the hypothalamus. In cachectic patients, populations of NPY/AgRP and POMC/CART neurons respond to systemic inflammatory mediators such as TNF-α, IL1-β and IL-6, which can induce an anorexigenic response [141]. Similarly, pro-inflammatory cytokines such as TNF-α and IL-1, which regulate leptin gene expression, play a central role in this inflammatory milieu. Although patients with cachexia have significantly lower leptin levels, this reduction does not lead to an increase in appetite or a decrease in energy expenditure [142]. On the contrary, the dysregulation of hypothalamic feedback, together with the action of cytokines such as IL-1, IL-6 and TNF-α produced by white adipose tissue (WAT), contributes to lipolysis and the loss of skeletal muscle mass that characterise the disease. Notably, leptin levels fall much more than expected based solely on fat mass reduction, which suggests an altered adipose tissue signalling in cachexia [132].

In addition to these alterations in the hypothalamic axis, our group showed that cachexia compromises the morphology of the CNS, primarily causing changes in the grey matter (GM) of patients. These alterations include modifications in regional volume pattern, functional connectivity, neuronal morphology, and neuroglial profile, while also inducing neuroinflammation. Such processes may contribute to the loss of homeostatic control and impaired information processing, as well as to the metabolic and behavioural disturbances commonly observed in human cachexia [143].

These processes, including increased lipolysis, enhanced lipid utilization, impaired adipogenesis and lipogenesis, IR, chronic inflammation, and fibrosis, accelerate fat loss and impair the metabolic function of AT, aggravating the progression of cachexia. Alterations in AT content and function are crucial factors in the evolution of cachexia, highlighting the importance of WAT remodelling in the development and progression of cancer cachexia [144]. Thus, WAT assumes a central role in sustaining chronic inflammation and in cachectic patients, both in the context of obesity and cancer-related cachexia (Table 1).

## 6. Discussion

Although obesity and cachexia may seem to represent opposing metabolic states, both conditions share underlying mechanisms involving systemic inflammation and metabolic dysfunction. These similarities, particularly in the context of adipose tissue (AT) dysregulation, provide insights into how seemingly contrasting conditions can converge on common pathways of energy imbalance and tissue degradation. 

The discussion on the interaction between obesity and cachexia highlights significant changes in WAT that contribute to systemic inflammation, altered metabolism, and IR. In obesity, visceral adipose tissue (vWAT) is particularly prone to inflammation and metabolic dysfunction. Hypertrophy, defined as the enlargement of adipocytes through lipid accumulation and differentiation of pre-adipocytes into mature adipocytes, occurs in vWAT in response to caloric excess. This is accompanied by increased infiltration of inflammatory immune cells, such as macrophages, primarily of the pro-inflammatory M1 phenotype. These macrophages secrete cytokines such as TNF-α and IL-6, exacerbating the inflammation eventually resulting in lipotoxicity, systemic inflammation and contributing to IR and metabolic syndromes [82]. In contrast, subcutaneous adipose tissue (sWAT) usually expands through hyperplasia (increased adipocyte number) rather than hypertrophy. However, excessive caloric intake can also induce inflammation in sWAT, compromising its metabolic function [75,145].

In cachexia, sWAT seems to be the major contributor to systemic inflammation and also presents profound changes in its architecture. Studies have shown that pre-adipocytes in cachexia display altered differentiation capacity, contributing to defective adipogenesis and increased fibrosis [130,136,146,147]. The presence of inflammatory cytokines in WAT plays a key role in the development of IR. In obese individuals, the imbalance between pro-inflammatory cytokines and anti-inflammatory factors such as adiponectin results in a state of low-grade chronic inflammation known as ‘metainflammation’ [148]. In cachexia, tumour-derived factors induce a systemic inflammatory response that accelerates the loss of muscle mass and fat, impairing metabolic homeostasis [149,150].

Lipogenesis enables adipocytes to convert glucose and fatty acids into triglycerides, a process that depends on lipoprotein lipase (LPL) and subsequent lipid re-esterification [151]. When this pathway is impaired, adipose tissue becomes dysfunctional, a feature observed in both obesity and cachexia.

Lipolysis, the breakdown of triglycerides in adipocytes, is affected in both obesity and cachexia, but with different outcomes. In obesity, increased lipolysis contributes to the formation of new fat depots, as free fatty acids released from hypertrophic adipocytes in vWAT activate inflammatory pathways and lead to ectopic fat deposition in organs such as the liver and muscles, worsening IR. In cachexia, chronic inflammation leads to increased lipolysis, but instead of promoting fat storage, it results in enhanced energy expenditure and rapid loss of fat stores, accelerating weight loss and metabolic imbalance [82,152].

**Figure 1:**
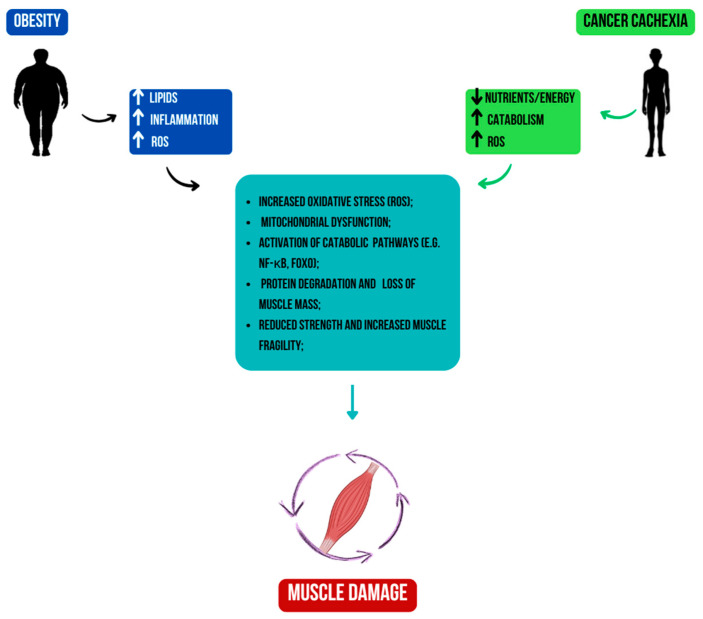
Increased oxidative stress in both conditions leads to mitochondrial dysfunction and the activation of catabolic pathways, such as NF-κB and FOXO, resulting in muscle atrophy and weakness.

Oxidative stress is another shared hallmark between obesity and cachexia. In both conditions, mitochondrial dysfunction leads to excessive production of reactive oxygen species (ROS), contributing to muscle wasting, IR, and systemic inflammation [153,154]. ROS can impair mitochondrial biogenesis, disrupt signalling pathways, and exacerbate cellular damage (Figure 1).

Despite the significant advances in understanding the metabolic and inflammatory pathways involved in both obesity and cachexia, many questions remain. The precise molecular crosstalk between adipose tissue depots and other tissues in these conditions is not fully understood.

**Figure 2:**
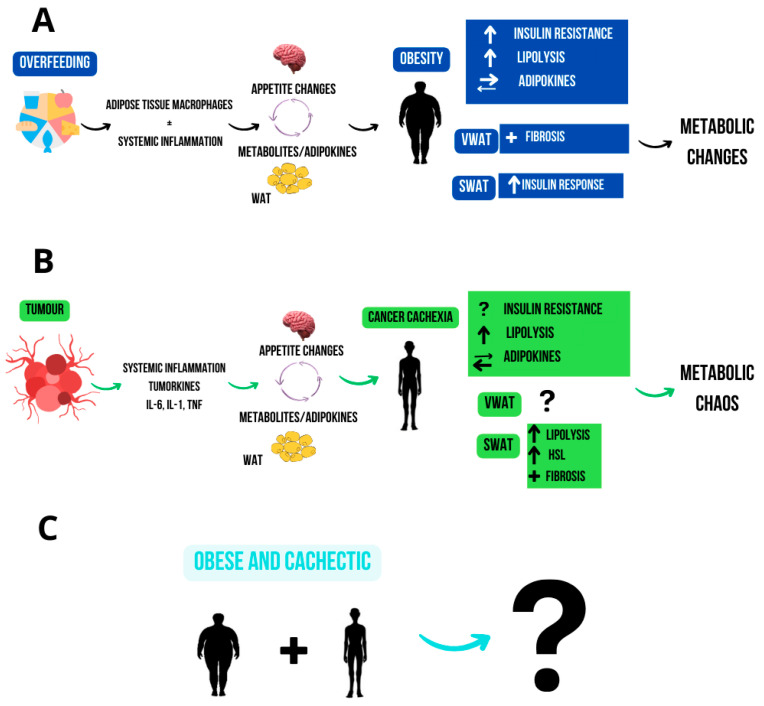
This image illustrates the metabolic outcomes of obesity and cancer cachexia. (**A**) Obesity leads to inflammation, IR, and increased lipolysis, particularly in visceral fat. (**B**) In cancer cachexia, tumour-driven inflammation causes metabolic dysfunction with Undefined impacts on fat tissues. (**C**) metabolic implications in individuals who are simultaneously obese and cachectic.

Interestingly, obesity and cachexia, traditionally viewed as opposites, can coexist (Figure 2). Prado et al. (2008) [155] demonstrated that obesity does not exclude the presence of neoplastic cachexia. The excess fat mass in obesity can obscure the clinical manifestations of cachexia, making it challenging to detect muscle loss when relying solely on weight loss for diagnosis. Cachexia in obese individuals is complex and multifactorial, with chronic inflammation likely playing a crucial role in the changes observed in adipose and muscle tissue seen in sarcopenic obesity. This understanding has reshaped paradigms in oncology, emphasising the need for precise body composition assessments rather than relying solely on BMI [156]. Advanced imaging techniques such as computerised tomography (CT), magnetic resonance imaging (MRI), dual-energy X-ray absorptiometry (DEXA), and ultrasound (USG) provide detailed analyses of lean and adipose tissue proportions [157]. These tools enable early detection of cachexia in obese individuals and have important implications for the diagnosis and management of cancer patients. Recognising the overlap between obesity and cachexia is clinically significant; in obese patients with cancer, cachexia can emerge as a severe complication, further exacerbating systemic inflammation and promoting a catabolic state that is difficult to reverse. Accurate assessment of body composition allows for more targeted interventions aimed at reducing inflammation and preserving muscle mass [158,159].

Of particular interest is myosteatosis, present both in cachexia as in obesity [160]. The degree and specificity of the alterations must be better understood, in order to clearly understand if obese patients with cancer cachexia will present synergistic effects or, rather, compensate for opposite regulatory mechanisms. 

## 7. Conclusions

This review highlights the converging metabolic alterations and inflammatory responses of obesity and cancer cachexia, particularly through the lens of WAT remodelling. Processes such as lipogenesis, lipolysis, oxidative stress, and adipokine secretion underline the complex adaptations of adipose tissue in both extremes of energy balance. Recognising these shared mechanisms provides valuable insight into the dual challenge of treating cachexia in patients with pre-existing obesity.

Lipogenesis is a critical metabolic process in which adipocytes convert glucose and free fatty acids into triglycerides for storage. This process depends on enzymes such as lipoprotein lipase (LPL), which hydrolyses circulating triglycerides into free fatty acids, and on re-esterification pathways that allow the incorporation of these fatty acids into lipid droplets [151]. Impaired lipogenesis contributes to adipose tissue dysfunction in both obesity and cachexia.

Lipolysis, the breakdown of triglycerides stored in adipocytes, is driven by the coordinated action of specific enzymes. The key enzymes involved in lipolysis are adipose triglyceride lipase (ATGL), which catalyses the initial step of triglyceride breakdown into diacylglycerol (DAG) and free fatty acids (FFA); hormone-sensitive lipase (HSL), which hydrolyses DAG into monoacylglycerol (MAG) and FFA; and monoacylglycerol lipase (MGL), which completes the process by breaking down MAG into glycerol and FFA [161].

In obesity, lipolysis is often dysregulated due to chronic IR and increased levels of inflammatory cytokines (such as TNF-α and IL-6), which impair insulin’s antilipolytic action (Sancar et al., 2024 [162]). Despite elevated lipolytic activity, the increased availability of FFA leads to enhanced re-esterification and fat storage, particularly in visceral adipose tissue (vWAT), contributing to ectopic fat deposition and metabolic dysfunction [101,163]

In contrast, in cachexia, increased lipolysis is driven primarily by tumour-derived factors (e.g., IL-6, TNF-α, and PTHrP) and systemic inflammation, leading to a net loss of fat mass rather than fat accumulation (Joshi & Patel 2022 [164]). The sustained activation of ATGL and HSL promotes an accelerated breakdown of fat stores, which exceeds the capacity for re-esterification, resulting in rapid fat depletion and metabolic imbalance [163,165].

Interestingly, lipid cycling—defined as the continuous breakdown and re-esterification of triglycerides—is increased in both obesity and cachexia but with distinct metabolic consequences. In obesity, increased lipid cycling contributes to inefficient fat storage and accumulation in non-adipose tissues (lipotoxicity). In cachexia, enhanced lipid cycling reflects a futile metabolic process that increases energy expenditure and accelerates tissue wasting [165].

The coexistence of these conditions underscores the need for further research into their common pathways and the development of personalised therapeutic strategies. Understanding these inflammatory and metabolic connections will help clinicians address the unique challenges posed by these coexisting conditions, ultimately improving patient outcomes and mitigating disruptions in a scenario which is even more frequent.

## Figures and Tables

**Table 1:** Comparing white adipose tissue deposits in obesity and cachexia.

Features	WAT in Obesity		WAT in Cachexia	
	Subcutaneous	Visceral	Subcutaneous	Visceral
Expansion of adipocytes	↑ Hyperplasia/hypertrophy	↑ Hyperplasia/hypertrophy	↓ Atrophy	↓ Atrophy
Inflammation	+	++	+++	++
Infiltration of immune cells	+	+++	+++	+
Adiponectin secretion	↑	↓	↑	↔
Leptin secretion	↔	↑	↓	↓
Insulin resistance	+	+++	↔	↔
Fibrosis	++	+	++	+
Impact of survival	+	++	+++	+

**Legend:** Symbols represent the intensity of the alterations: + mild, ++ moderate, +++ marked, ↑ increase, ↓ decrease, ↔ no change.

## Data Availability

No new data were created or analyzed in this study. Data sharing is not applicable to this article.
